# Vaginal birth after caesarean versus elective repeat caesarean delivery after one previous caesarean section: a cost-effectiveness analysis in four European countries

**DOI:** 10.1186/s12884-018-1720-6

**Published:** 2018-04-11

**Authors:** Maaike Fobelets, Katrien Beeckman, Gilles Faron, Déirdre Daly, Cecily Begley, Koen Putman

**Affiliations:** 1 I-CHER (Interuniversity Centre for Health Economics Research), Faculty of Medicine and Pharmacy, Vrije Universiteit Brussels, Laarbeeklaan 103, 1090 Brussels, Belgium; 2 Department of Public Health, Faculty of Medicine and Pharmacy, Vrije Universiteit Brussel, Laarbeeklaan 103, 1090 Brussels, Belgium; 3 Department of Nursing and Midwifery, Nursing and Midwifery research group, Universitair Ziekenhuis Brussel, Laarbeeklaan 103, 1090 Brussels, Belgium; 4 Department of Obstetrics, Universitair Ziekenhuis Brussel, Vrije Universiteit Brussel, Laarbeeklaan 101, 1090 Brussels, Belgium; 5 School of Nursing and Midwifery, Trinity College Dublin, 24 D’Olier Street, Dublin, D02 T283 Ireland; 6 Institute of Health and Care Sciences, Sahlgrenska Academy, University of Gothenburg, Gothenburg, Sweden

**Keywords:** Cost-effectiveness analysis, Elective repeat caesarean delivery (ERCD), Vaginal birth after caesarean (VBAC)

## Abstract

**Background:**

The OptiBIRTH study incorporates a multicentre cluster randomised trial in 15 hospital sites across three European countries. The trial was designed to test a complex intervention aimed at improving vaginal birth after caesarean section (VBAC) rates through increasing women’s involvement in their care. Prior to developing a robust standardised model to conduct the health economic analysis, an analysis of a hypothetical cohort was performed to estimate the costs and health effects of VBAC compared to elective repeat caesarean delivery (ERCD) for low-risk women in four European countries.

**Methods:**

A decision-analytic model was developed to estimate the costs and the health effects, measured using Quality Adjusted Life Years (QALYs), of VBAC compared with ERCD. A cost-effectiveness analysis for the period from confirmation of pregnancy to 6 weeks postpartum was performed for short-term consequences and during lifetime for long-term consequences, based on a hypothetical cohort of 100,000 pregnant women in each of four different countries; Belgium, Germany, Ireland and Italy. A societal perspective was adopted. Where possible, transition probabilities, costs and health effects were adapted from national data obtained from the respective countries. Country-specific thresholds were used to determine the cost-effectiveness of VBAC compared to ERCD. Deterministic and probabilistic sensitivity analyses were conducted to examine the uncertainty of model assumptions.

**Results:**

Within a 6-week time horizon, VBAC resulted in a reduction in costs, ranging from €3,334,052 (Germany) to €66,162,379 (Ireland), and gains in QALYs ranging from 6399 (Italy) to 7561 (Germany) per 100,000 women birthing in each country. Compared to ERCD, VBAC is the dominant strategy in all four countries. Applying a lifetime horizon, VBAC is dominant compared to ERCD in all countries except for Germany (probabilistic analysis, ICER: €8609/QALY). In conclusion, compared to ERCD, VBAC remains cost-effective when using a lifetime time.

**Conclusions:**

In all four countries, VBAC was cost-effective compared to ERCD for low-risk women. This is important for health service managers, economists and policy makers concerned with maximising health benefits within limited and constrained resources.

**Electronic supplementary material:**

The online version of this article (10.1186/s12884-018-1720-6) contains supplementary material, which is available to authorized users.

## Background

Rates of caesarean section have increased steadily in Europe [1, 2], and in many countries worldwide [3] in the past three decades. This rise in rates, and the wide variation in rates between and within countries has led to research exploring the factors contributing to the increase, and into the comparative risks of giving birth by caesarean section and vaginally [4]. One factor contributing to the increasing rates of caesarean section is elective repeat caesarean delivery (ERCD), a term used to indicate that the caesarean birth is deemed necessary prior to the start of labour, and is not performed as an emergency procedure. Nowadays, more women who had one previous caesarean section are being offered the possibility of having a subsequent birth vaginally. Studies have shown that vaginal birth after caesarean (VBAC) for low-risk women is associated with benefits compared to an ERCD [4–7]. Coupled with this is a growing interest in costs, and results from health economic analyses have demonstrated the cost effectiveness of VBAC compared to ERCD [8–12]. VBAC was found to be a dominant strategy, i.e. less expensive and more effective than the ERCD strategy [9–11, 13]. The underlying reasons for this finding is that VBAC results in a reduced length of hospital stay postpartum and is associated with fewer long-term maternal morbidities such as hysterectomy, placenta praevia and mortality [9, 11, 14–18]. Conversely, VBAC was associated with higher rates of uterine ruptures, long term neonatal morbidities such as cerebral palsy, and neonatal death [14, 16, 17, 19–21].

Internationally, several health economic analyses have been performed by using different costing techniques i.e. different perspectives, variation in perinatal outcomes accounted for in the analyses and differences in time horizons [8–12, 22]. The health services perspective is the most frequently used [7]. However, this perspective has some limitations; it excludes important items such as direct costs borne by women and their families, indirect costs associated with welfare losses and productivity losses. Perinatal outcomes included in health economic analyses vary considerably, both for the mother and newborn. Some studies do not include the neonatal outcomes [23], though the need to include these outcomes has been demonstrated previously [5, 6, 11, 13]. Including some perinatal outcomes in health economic analysis on mode of birth requires a longer time horizon than the commonly used perinatal care episode. Some longer-term conditions affecting the newborn, e.g. cerebral palsy, are associated with significant costs and major losses of quality of life for both the newborn/child and the parents. Conditions affecting the woman in subsequent pregnancies, e.g. placenta accreta or praevia are a cause of maternal morbidity of women with multiple ERCDs. Including these outcomes would mean using a lifetime horizon. However, using a more standardised health economic analysis model for one prior low transverse caesarean delivery, with a core set of outcomes and a lifetime horizon, would already increase the comparability across models.

Clinicians and health policy makers, concerned about the rising rates of caesarean sections, are seeking ways of stopping these rates from rising further, and reducing the number of caesarean sections being performed [7]. An example of the growing interest in VBAC is the OptiBIRTH project [24, 25], a part of which is an international multicentre trial that aims to increase the proportion of women having VBAC by increasing women-centred care and facilitating women’s empowerment in their choice of birth in three countries, Germany, Ireland and Italy. As a part of this project, a health economic evaluation was planned to evaluate the cost effectiveness across these three countries. Performing a health economic analysis on mode of birth is challenging, especially in the context of a multi-country study, because of the variability in the content of usual care, the organisation of the healthcare system and the availability of appropriate data on costs [9]. Using a more uniform health economic model applied in a multi-country context would be beneficial, increasing the comparability and external validation of the results. To our knowledge, no such uniform health economic model comparing VBAC with ERCD, from societal perspective, with a time horizon starting from pregnancy recognition up to 6 weeks postpartum, and including the costs and consequences for both the mother and newborn, has been published. The model was conducted for four European Union (EU)-countries, Germany, Ireland, Italy where the trial was being conducted, and Belgium, where the health economic analysis was being conducted.

## Methods

### Model

**Fig. 1:**
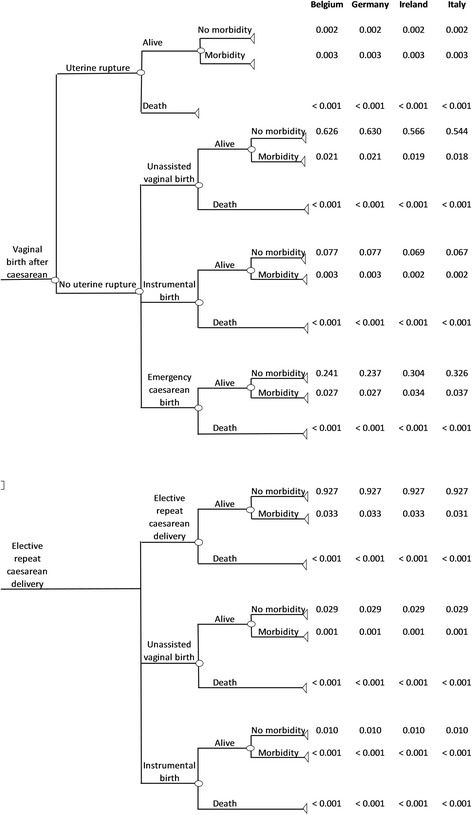
Decision tree comparing vaginal birth after caesarean with elective repeat caesarean delivery

A decision analytic model was developed to compare the costs and consequences of VBAC and ERCD. The decision tree (Fig. 1) was built based on previously published models and expert opinion (GF) [7, 9]. A hypothetical cohort of 100,000 women was used for each country (Belgium, Germany, Ireland and Italy). Women included in the model were low-risk (i.e. without pre-existing medical conditions or risk factors), with a singleton pregnancy, at term gestation who had one previous caesarean section that was performed using low uterine transverse incision. The assumption is that in this cohort of women, there are no contraindications to VBAC [26] at time of admission for giving birth. The time horizon of the model was from pregnancy recognition up to 6 weeks postpartum for short-term consequences and a lifetime horizon was applied for long-term consequences. The decision tree consisted of two main arms, VBAC and ERCD. Women in the VBAC arm may have a successful VBAC resulting in an unassisted vaginal birth or instrumental vaginal birth. Women in this arm may also experience an emergency caesarean section or uterine rupture. In the ERCD arm, women may have an ERCD, and a small proportion of women might go into labour spontaneously resulting in an unassisted vaginal birth or instrumental vaginal birth. Women planning an ERCD may also suffer uterine rupture but, as it occurs only rarely, we have not included it in the model. In all cases, women could have no morbidity, suffer a morbidity, or die. To account for maternal morbidity, the maternal outcomes included were: uterine rupture, endometritis, peripartum hysterectomy, blood transfusion, thrombotic events, operative injury and wound complications [11, 15–21, 27–30]. Neonatal health outcomes were not built directly into the decision tree but accounted for via the health state of the women (see below) [11]. A neonate could be born alive and have no morbidity, suffer a morbidity or die. In cases of neonatal morbidity, the following neonatal outcomes were evaluated: hypoxic ischemic encephalopathy (HIE), sepsis and respiratory conditions including transient tachypnoea of the newborn (TTN) and respiratory distress syndrome (RDS) [5, 6, 11, 17, 20, 31–38]. Cerebral palsy was included in the model as a long-term consequence of HIE, assuming that 12% of the infants would finally be diagnosed with cerebral palsy [11, 39]. Country-specific data on the VBAC success rates were available for Germany (74%), Ireland (66%) and Italy (64%), and the overall European success rate was used for Belgium (73%) [9, 40–42]. All other probabilities, including maternal and neonatal morbidities and mortality, were based on data obtained from the literature, and were identical for all countries (Additional file 1: Table S2) [5, 6, 9, 11, 15–21, 27–38, 43, 44]. Based on the probability data, the probability per arm was calculated by multiplying the probability of each branch to calculate the final probability of the endnodes e.g. for unassisted vaginal birth: probability of unassisted vaginal birth (VBAC success rate * proportion of unassisted vaginal birth * probability to be alive * probability to have no morbidities) (Fig. 1).

### Costs and utilities

The analysis was performed from a societal perspective and included maternal and neonatal health outcomes and costs. The costs included direct medical costs for the ante-, peri- and postnatal periods and non-medical costs and indirect costs. An overview of all costs and their respective sources can be found in Table 1 and in Additional file 1: Tables S1 and S2. For each country, antenatal resource usage calculations were based on the national antenatal/neonatal guidelines and were validated by an expert in each country. One extra consultation with an anaesthesiologist was included when an ERCD was planned. Ambulatory costs, i.e. costs for outpatient care, were obtained from country specific ambulatory tariff lists and linked to resource use. Inpatient costs of the mother and newborn were calculated per health-state based on country-specific Diagnostic Related Groups (DRG) data. Two extra visits with an obstetrician and paediatrician were included in cases of short-term maternal and/or neonatal morbidities, and direct non-medical costs (i.e. travel costs) were also included. The average distance to ambulatory centres and hospitals was multiplied by the unit cost for transportation. The cost of HIE was estimated as half of the yearly cost of CP, for 2 years [11]. For long-term consequences such as cerebral palsy and maternal and neonatal mortality, lifetime costs were calculated including the lifetime societal cost for cerebral palsy for 50 years [13] and lifetime productivity loss for maternal and neonatal mortality. National discount rates for costs and utilities were used: Belgium (3%, 1.5%), Germany (5%, 5%), Ireland (5%, 5%) and Italy (3%, 3%) [45–48]. The Harmonized Indices of Consumer Prices (HICP) - health were used to convert all costs to 2016 euros [49].

**Table 1:** Cost estimates, €^a^

	Belgium	Germany	Ireland	Italy
Antenatal care				
Intention: vaginal birth after caesarean	535	791	1656	1017
Intention: elective repeat caesarean delivery	563	826	1844	1095
Mode of birth				
Unassisted vaginal birth				
Without complications	2948	1745	2337	1315
With complications	3363	2394	4065	1674
Instrumental vaginal birth				
Without complications	3363	2049	3063	2956
With complications	4790	2394	4065	2956
Elective repeat caesarean delivery				
Without complications	4083	2659	4805	2163
With complications	5105	3180	6242	2876
Emergency caesarean section				
Without complications	5105	3661	4805	2163
With complications	8261	3903	6242	2876
Uterine Rupture				
Without complications	5105	3903	4805	2876
With complications	8261	3903	6242	2876
Neonatal HIE/CP (six-week time horizon)	12,347	7587	10,275	4052
Neonatal HIE (first 2 years)	16,926	16,423	17,093	15,250
Neonatal CP (lifetime)	846,309	821,171	854,688	762,516
Neonatal Sepsis	7266	19,413	15,408	4052
Neonatal respiratory conditions				
Transient tachypnoea	3007	788	1485	579
Respiratory distress syndrome	11,392	19,413	15,408	13,478
Neonatal mortality	4712	7198	7036	5579
Postnatal care				
Without complications	182	622	135	308
With complications	329	782	885	643
Productivity loss (per year)	48,511	46,373	46,607	35,272

*HIE* hypoxic ischemic encephalopathy, *CP* cerebral palsy

^a^Currency in 2016 euros (€) [49]

Total costs, travel costs included. More detailed costs and their sources are described in Additional file 1: Table S1 and S2

Quality-adjusted life years (QALYs) were used to measure outcomes for the different health states (Table 2 and Additional file 1: Table S2). The country-specific utilities for normal health were considered as the base-case utility [50, 51]. Ireland did not have country-specific values available, and UK utility values were used. Utility values during the antenatal period were assumed to be equal for both groups, VBAC and ERCD. Thereafter, utilities were calculated for each maternal and neonatal outcome separately by reducing the country-specific utilities for normal health with the corresponding disutility of the maternal and neonatal outcome. No country-specific disutilities could be obtained, consequently, the disutility values were based on data obtained from the literature [9, 11, 12, 52].

**Table 2:** Utilities by country and disutilities by mode of birth or outcome^a^

Country	Utility (per day^b^)	Duration (days)	References
Belgium	0.98	42	[51]
Germany	0.99	42	[50]
Ireland	0.99	42	[50]
Italy	0.99	42	[50]
Mode of birth/outcome	Disutility (per day)	Duration (days)	References
Successful vaginal birth after caesarean	0.41	7	[9]
Elective repeat caesarean delivery	0.58	21	[9]
Emergency caesarean birth	0.58	21	[9]
Hysterectomy	0.58	21	[9]
Uterine rupture	0.58	21	[9]
Endometritis	0.38	14	[9]
Blood transfusion	0.41	7	[9]
Thrombotic events	0.41	14	Assumption
Operative injury	0.53	21	[9]
Wound complication	0.53	21	Assumption
Well (no adverse outcome)	0	42	[12]
HIE	0.75	21	[11]
CP	0.53	All	[11]
Sepsis	0.01	42	[52]
Respiratory conditions	0.01	14	[52]
Maternal/neonatal death	1	All	[12]

*HIE* hypoxic ischemic encephalopathy, *CP* cerebral palsy

^a^ parameter distributions can be found in Additional file 1: Table S2

^b^ daily utilities were derived from annual utilities

The primary outcome was the incremental cost-effectiveness ratio (ICER) per QALY gained using the expected costs and QALYs gained by using the following formula:

$$ \mathrm{ICER}=\frac{\left(\mathrm{Expected}\ \mathrm{Cost}\ \mathrm{VBAC}-\mathrm{Expected}\ \mathrm{Cost}\ \mathrm{ERCD}\right)}{\left(\mathrm{Expected}\ \mathrm{Utility}\ \mathrm{VBAC}-\mathrm{Expected}\ \mathrm{Utility}\ \mathrm{ERCD}\right)} $$

The cost-effectiveness threshold was country dependent. For Belgium, Germany and Italy, the gross domestic product (GDP) per capita was used, with an ICER of €36,633/QALY, €37,719/QALY and €27,219/QALY respectively [53]. The cost-effectiveness threshold for Ireland was historically set at €45,000/QALY [9]. Additionally, the incremental net monetary benefit (INMB) between VBAC versus ERCD was calculated applying various thresholds in order to evaluate the cost-effectiveness (Additional file 1: Figure S2).

### Sensitivity analysis

To identify the uncertainty of parameters and the robustness of the results of the base-case model, sensitivity analyses were performed. Both one-way and probabilistic sensitivity analyses were conducted on the input parameters for all costs, probabilities and utilities used in the model. In the one-way sensitivity analysis, input parameters were varied by 30% of their initial value, and parameters with the highest impact are presented in Tornado diagrams. Simulations were conducted by using beta and Dirichlet distributions for probabilities and utilities, and gamma distributions for costs. A probabilistic sensitivity analysis was performed using Monte Carlo simulations with 5000 iterations.

## Results

### Base-case scenario analysis

The base-case analysis comparing VBAC with ERCD for this hypothetical cohort resulted in an average of 32,312 fewer caesarean sections per 100,000 women in each of the four countries (Additional file 1: Table S3). The ICER results of the deterministic analysis for a 6-week time horizon demonstrated that VBAC was a dominant strategy in all four countries, which means less costly and more effective than ERCD. This results in a negative ICER of -€441/QALY for Germany, −€2035/QALY for Belgium, −€3042/QALY for Italy and -€9891/QALY for Ireland (Table 3). When considering a lifetime time-horizon, VBAC was a dominant strategy in Ireland (−€96,753/QALY) and Italy (−€15,965/QALY). Compared to ERCD, VBAC remained a cost-effective strategy, which means slightly more costly and more effective, for Belgium (€3669/QALY) and Germany (€12,817/QALY). The overall maternal morbidity rate was higher in the VBAC arm compared to the ERCD arm (5.64% versus 3.25%) due to the morbidity related to performing emergency caesarean sections. The neonatal morbidity rate, 6.82% for VBAC and 5.14% for ERCD, and mortality rate, 0.02% for VBAC and 0.00% for ERCD, were higher in the VBAC arm due to the higher amount of complications in case of an emergency caesarean section or uterine rupture. The overall maternal mortality rate was comparable for both groups (0.01% and 0.00% in both groups).

**Table 3:** Cost-effectiveness results of vaginal birth after caesarean versus elective repeat caesarean per woman, €^a^

Model	Vaginal birth after caesarean		Elective repeat caesarean delivery		ICER	Total costs (€)	Total utilities (QALY)
	Cost (€)	Utility (QALY)	Cost (€)	Utility (QALY)	(€/QALY)		
Six-week time horizon							
Belgium						−15,271,888	7503
Deterministic	4862	0.84	5167	0.69	− 2035		
Probabilistic	4852	0.84	5171	0.69	− 2127		
Germany						−3,334,052	7561
Deterministic	4921	0.85	4988	0.70	−441		
Probabilistic	4921	0.85	4985	0.70	−423		
Ireland						−66,162,379	6689
Deterministic	6056	0.84	7379	0.70	− 9890		
Probabilistic	6057	0.84	7380	0.70	−9886		
Italy						−19,465,446	6399
Deterministic	4167	0.83	4556	0.70	−3042		
Probabilistic	4163	0.83	4552	0.70	− 3026		
Litetime time horizon							
Belgium						1,386,210	376
Deterministic	5281	34.79	5254	34.79	3669		
Probabilistic	5239	34.79	5251	34.77	− 596		
Germany						8,481,119	662
Deterministic	5222	19.12	5052	19.11	12,817		
Probabilistic	5147	19.12	5051	19.10	8609		
Ireland						−53,955,166	558
Deterministic	6363	18.64	7442	18.63	−96,753		
Probabilistic	6363	18.64	7442	18.63	−60,905		
Italy						−6,558,036	411
Deterministic	4488	26.44	4619	26.43	−15,965		
Probabilistic	4414	26.44	4618	26.42	−10,633		

*QALY* quality adjusted life year, *ICER* incremental cost-effectiveness ratio

^a^ Currency in 2016 euros (€) [49]

The costs and QALYs saved differ between the four countries. Within a 6-week time horizon, the total costs saved (per 100,000 women) were €3,334,052 in Germany, €15,271,888 in Belgium, €19,465,446 in Italy and €66,162,379 in Ireland and the total number of QALYs were 6399 in Italy, 6689 in Ireland, 7503 in Belgium and 7561 in Germany more per 100,000 women. When considering lifetime costs and consequences, the total cost of the VBAC strategy was €1,386,210 in Belgium and €8,481,119 in Germany with 376 QALYs and 662 QALYs more in Belgium and Germany. The total costs saved were €53,955,166 in Ireland and €6,558,036 in Italy and the total number QALYs were 558 in Ireland and 411 in Italy.

### Sensitivity analyses

**Fig. 2:**
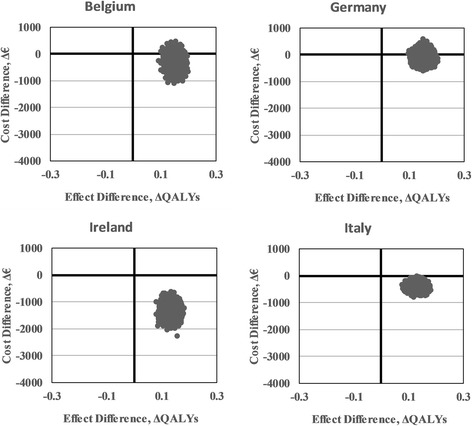
Cost-effectiveness planes (6 weeks time horizon)**.** Distribution of incremental costs and outcomes of vaginal birth after caesarean versus elective repeat caesarean delivery for Belgium, Germany, Ireland and Italy. 5000 Monte Carlo simulations were conducted for every country. QALY, quality adjusted life year

The results of the one-way sensitivity analysis showed the robustness of the models when varying the input parameters. Overall, the model was robust, i.e. varying parameters by the sensitivity analysis did not result in large variations of the ICERs. Four of the most influential drivers on the ICERs were similar for all four countries: the costs of antenatal care in both ERCD and VBAC, cost of an ERCD and unassisted vaginal birth without complications. For Belgium, Ireland and Italy, the (dis)utility associated with emergency caesarean birth was found to be sensitive variables for the ICER result. The probability of an unassisted vaginal birth was sensitive for Belgium, Germany and Italy. For the two latter countries, the disutility of an ERCD was also a sensitive variable. For Germany, the model was also sensitive for the probability of an ERCD and for the disutility of unassisted vaginal birth in Ireland (Additional file 1: Figure S2). The probabilistic sensitivity analysis resulted in a mainly dominant ICER with a reduction of the costs and increased effects when comparing VBAC with ERCD. The results differed between countries, ranging from -€423/QALY in Germany to -€9886/QALY in Ireland for a 6 week time horizon (Table 3), and from €8609/QALY in Germany to -€60,905 in Ireland for a lifetime time horizon. The most favourable ICER was for Ireland which can be explained by the high incremental cost of an ERCD compared to a vaginal birth. The lowest ICER was for Germany, as a result of the high probability and cost of neonatal sepsis in case of an emergency caesarean section or uterine rupture. The Monte Carlo simulations (6-week time horizon) are graphically presented as cost-effectiveness planes (Fig. 2), and are mainly plotted in the south-east quadrant for all four countries. For Belgium and Germany 7.34% and 34.50% of all simulations were plotted in the north-east quadrant, with the latter showing VBAC to be slightly more expensive compared to ERCD. All simulations remained far below the national cost-effectiveness thresholds [53] and the likelihood of cost-effectiveness at the national thresholds were 100% for all four countries. For the lifetime model, VBAC was the preferred strategy for 98.66% (Belgium) and 100.00% (Germany, Ireland and Italy) of all Monte-Carlo simulations.

## Discussion

We developed a standardised theoretical decision analytic model for four EU-countries comparing two strategies, attempting a VBAC and a planned ERCD. This model was adjusted to take into account more realistic clinical scenarios compared with previous health economic models, including the possibility of having a vaginal birth even when an ERCD had been planned. The results from these analyses confirm the cost-effectiveness of VBAC compared to ERCD in low-risk women. The probability of VBAC being cost-effective was almost 100% for the 6 weeks and lifetime time horizon for all four countries, and resulted in fewer caesarean sections.

Comparing these results with previously published health economic evaluations is difficult because of the variation in the models used, and the probabilities, utilities and costs included in these models [11]. We found four studies comparing VBAC with ERCD following one previous caesarean section of which one was European [9] and three from the United States of America [8, 10, 12]. Additionally, two cost-effectiveness studies compared VBAC with ERCD after one previous caesarean section over a lifetime horizon, and considered outcomes from subsequent pregnancies [11, 13]. All studies found the VBAC strategy to be cost-effective. Fawsitt et al.’s study [9], based on a hypothetical cohort in Ireland, found VBAC to be a dominant strategy resulting in an ICER -€15,773/QALY, comparable to our result of -€9886/QALY. However, their study included a limited number of maternal outcomes, e.g. thrombotic events and wound complications were not taken into consideration, and no neonatal outcomes (and associated costs) were included. The remaining health economic analyses included neonatal outcomes [8, 10–12]. Gilbert et al. [10] found that VBAC was a dominant strategy compared to ERCD and showed savings of $139 million, and gains of 1703 QALYs per 100,000 women. Grobman et al. [12] and Chung et al. [8] did not report the total number of QALYs gained; their cost-effectiveness analysis estimated $179 million saved per 100,000 women and $112,023/QALY respectively. Gilbert’s et al. [11] lifetime cost-effectiveness analysis resulted in $164 million saved and 500 QALYs gained per 100,000 women, with an ICER of $-328,200/QALY. The lifetime cost-effectiveness study of Wymer et al. [13] showed VBAC to be a dominant strategy compared to ERCD with an incremental cost and effectiveness of -$4700,00 and 0.073 QALYs per patient (mother and neonate) for the sixth delivery. Findings from our study of VBAC being cost-effective are comparable with previous studies [9–11]. When incorporating a lifetime time horizon, the VBAC strategy was found to be more effective for all four countries but also slightly more costly for Germany. Previous studies showed the VBAC strategy to become less costly and more effective when including more subsequent deliveries [11, 13]. For all four countries and for both time horizons (6 weeks and lifetime), the VBAC strategy was cost-effective compared to ERCD, and the ICER results remained far below the national cost-effectiveness thresholds.

To the best of our knowledge, our model is the first to be used in multi-country context. We performed the health economic analysis from a societal perspective by including direct medical costs and non-medical costs, such as costs borne by the women, and indirect costs such as productivity loss. This societal approach includes costs related to the society’s welfare, whereas the health services perspective considers only direct medical costs. We included costs and health effects for all three episodes of perinatal care, the ante-, peri- and postnatal periods for both the mother and newborn. Therefore, we believe this model is more comprehensive and better reflects the clinical situation in real life compared to previous models. All costs included in our model were country specific and, where possible, based on national data sources. Neonatal outcomes were included because mode of birth can impact some adverse neonatal outcomes such as cerebral palsy and sepsis. In addition, we developed a standardised model for application in all four countries. Our model builds on previously published models and offers a unique contribution to performing international health economic analyses. We have included all the model’s input parameters, thus giving other researchers the opportunity to test and use our model for future health economic evaluations in other countries which, ultimately, will increase the comparability of study results.

This study has limitations, primarily because our findings are based on a hypothetical cohort. However, wherever possible, we used country-specific probabilities, particularly for variables described as important in previous studies [8]. Health-effects were measured as QALYs, and country specific QALYs of the general population were reduced with the corresponding disutility of the maternal and neonatal outcome. In our method of calculating disutilities, we consider the mode of birth and neonatal outcome to be independent. As a consequence, this may lead in some scenarios to an over/under estimation of the disutilities due to double counting health effects. In our opinion, there is no clear method available to overcome this methodological issue. Further studies are needed, which attempt to disentangle the health effects on the mother and child, and give guidance on how the health effects of neonates can be considered separately in health economic evaluations. While all costs included in our model were country specific, for some costs, such as the in-patient DRG costs, we were unable to find a specific DRG cost for every arm of the decision tree. Some countries such as Italy and Ireland had only two DRGs for a caesarean section, one with and one without complications. Therefore, the cost of an ERCD or emergency caesarean, could only be distinguished based on the appearance of complications. We considered these uncertainties in the deterministic and probabilistic sensitivity analyses and found they did not have a high impact on the estimated ICER. In addition, we limited our cohort to women with only one previous caesarean section and included only the major short and long-term consequences.

The main strength of this study is the development of a standardised health economic analysis model which can be used for cost-effectiveness studies of VBAC versus ERCD in an international context. Other researchers may consider developing and advancing this model, and including more subsequent deliveries and more long-term consequences.

## Conclusions

This model is the first health economic analysis model that can be used to calculate the cost-effectiveness of VBAC and ERCD in an international context. The results of this study suggests that, compared to ERCD, VBAC is cost-effective in Belgium, Germany, Ireland and Italy. This model, and its underlying methods and assumptions can be applied to more EU-countries, or even beyond. This model can easily be used by researchers to calculate the cost-effectiveness of new strategies comparing VBAC with ERCD by inputting probability, costs and utility data collected during the study.

## Additional file

Additional file 1: Additional tables and files including an overview of resource use, costs and model parameters used in the models; overview of the distribution over maternal and infant outcomes per 100,000 women by mode of birth, by country; results of the incremental net monetary benefit per country; country specific tornado diagrams as a result of the one-way sensitivity analysis. **Table S1**. Healthcare utilisation and unit costs antenatal and postnatal care. **Table S2**. Model parameters and distributions probabilistic sensitivity analysis. **Table S3**. Distribution mode of birth by country. **Figure S1**. Incremental Net Monetary Benefit (lifetime horizon). **Figure S2**. Country specific tornado diagrams as a result of the one-way sensitivity analysis (6-week time horizon) (DOC 1501 kb)

## Abbreviations

CP: Cerebral palsy
DRG: Diagnostic related groups
ERCD: Elective repeat caesarean delivery
EU: European Union
HICP: Harmonized indices of consumer prices
HIE: Hypoxic ischemic encephalopathy
ICER: Incremental cost-effectiveness ratio
QALY: Quality adjusted life years
VBAC: Vaginal birth after caesarean
