# Supplementary material for: Vaginal birth after caesarean versus elective repeat caesarean delivery after one previous caesarean section: a cost-effectiveness analysis in four European countries
Source: BMC Pregnancy Childbirth. 2018 Apr 11;18:92. doi: 10.1186/s12884-018-1720-6 (PMC5896042; doi:10.1186/s12884-018-1720-6)
Supplement: Supplementary file 1 — Additional tables and files including an overview of resource use, costs and model parameters used in the models; overview of the distribution over maternal and infant outcomes per 100,000 women by mode of birth, by country; results of the incremental net monetary benefit per country; country specific tornado diagrams as a result of the one-way sensitivity analysis. Table S1. Healthcare utilisation and unit costs antenatal and postnatal care. Table S2. Model parameters and distributions probabilistic sensitivity analysis. Table S3. Distribution mode of birth by country. Figure S1. Incremental Net Monetary Benefit (lifetime horizon). Figure S2. Country specific tornado diagrams as a result of the one-way sensitivity analysis (6-week time horizon) (DOC 1501 kb) [file 12884_2018_1720_MOESM1_ESM.doc]

**Additional file**

| **Table S1.** Healthcare utilisation and unit costs antenatal and postnatal care | | | | | | | | | | | | |
| --- | --- | --- | --- | --- | --- | --- | --- | --- | --- | --- | --- | --- |
|  | **Belgium** | |  | **Germany** | |  | **Ireland** | |  | **Italy** | |  |
|  | **Healthcare utilisation** | **Unit cost**  **(€)Ɨ** | **Ref** | **Healthcare utilisation** | **Unit cost**  **(€)Ɨ** | **Ref** | **Healthcare utilisation** | **Unit cost**  **(€)Ɨ** | **Ref** | **Healthcare utilisation** | **Unit cost**  **(€)Ɨ** | **Ref** |
| **ANTENATAL CARE** | |  |  |  |  |  |  |  |  |  |  |  |
| **Visits with healthcare providers** |  |  |  |  |  |  |  |  |  |  |  |  |
| Obstetrician | 7 | 24.71 |  | 11 | 30.93 |  | 2 | 173.87 |  | 6 | 76.52 |  |
| Midwife |  |  |  | 1 | 36.11 |  | 3 | 92.37 |  |  |  |  |
| General Practitioner |  |  |  |  |  |  | 6 | 29.26 |  |  |  |  |
| Anaesthesiologist  (extra visit ERCD arm) | 1 | 20.77 |  | 1 | 25.33 |  | 1 | 173.87 |  | 1 | 72.37 |  |
| **Diagnostic tests** |  |  |  |  |  |  |  |  |  |  |  |  |
| Ultrasound scans | 3 | 23.10 |  | 3 | * |  | 2 | 173.87 |  | 3 | 103.85 |  |
| Urine sediment/culture | 1 | 23.42 |  |  |  |  |  |  |  | 6 | 4.37 |  |
| Urine dipstick | 7 | 0.50 |  | 11 | 0.50 |  | 12 | 0.50 | Ass | 6 | 0.50 | Ass |
| Vaginal culture  (Group B streptococcal screening) | 1 | 10.12 |  | 1 | * |  |  |  |  | 1 | 13.11 |  |
| Gestational Diabetic Screening  (OGTT 75g glucose) | 1 | 22.20 |  | 1 | 12.61 |  |  |  |  | 1 | 6.55 |  |
| **Table S1 continued** |  |  |  |  |  |  |  |  |  |  |  |  |
|  | **Belgium** |  |  | **Germany** |  |  | **Ireland** |  |  | **Italy** |  |  |
|  | **Healthcare utilisation** | **Unit cost (€)Ɨ** | **Ref** | **Healthcare utilisation** | **Unit cost**  **(€)Ɨ** | **Ref** | **Healthcare utilisation** | **Unit cost**  **(€)Ɨ** | **Ref** | **Healthcare utilisation** | **Unit cost**  **(€)Ɨ** | **Ref** |
| Down Screening blood sample (PAPP-A and β-HCG) | 1 | 73.28 |  |  |  |  |  |  |  |  |  |  |
| Indirect coombs | 1 | 8.09 |  | 1 | 8.30 |  |  |  |  | 2 | 7.65 |  |
| Rubella IgG | 1 | 6.87 |  | 1 | 10.06 |  | 1 | 9.03 |  | 1 | 10.93 |  |
| Rubella IgM | 1 | 6.87 |  | 1 | 10.06 |  | 1 | 30.09 |  | 1 | 10.93 |  |
| Toxoplasmosis IgG | 1 | 6.87 |  |  |  |  | 1 | 25.58 |  | 1 | 10.93 |  |
| Toxoplasmosis IgM | 1 | 7.28 |  |  |  |  | 1 | 22.57 |  | 1 | 10.93 |  |
| Liver transaminases  (AST/ALT (SGOT/SGPT)) |  |  |  |  |  |  |  |  |  | 1 | 4.37 |  |
| VDRL | 1 | 6.87 |  | 1 | 4.57 |  | 1 | 18.81 |  | 1 | 8.75 |  |
| Anti HIV | 1 | 6.87 |  | 1 | 4.25 |  | 1 | 10.53 |  | 2 | 18.58 |  |
| Anti HCV |  |  |  |  |  |  |  |  |  | 1 | 14.21 |  |
| HbsAg | 1 | 6.87 |  | 1 | 5.71 |  | 1 | 22.57 |  | 1 | 14.21 |  |
| Blood group and Rhesus factor | 1 | 6.05 |  | 2 | 8.62 |  | 1 | 47.19 |  | 1 | 9.84 |  |
| Blood group C,c, E and e |  |  |  | 2 | 4.25 |  |  |  |  | 1 | 5.47 |  |
| Complete blood count** | 2 | 12.75 |  | 7 | * |  | 2 | 2.18 |  |  |  |  |
| Chlamydia trachomatis |  |  |  | 1 | 8.82 |  |  |  |  |  |  |  |
| Cytomegalovirus |  |  |  |  |  |  | 1 | 45.89 |  |  |  |  |
| Herpes | 1 | 21.14 |  |  |  |  | 1 | 48.14 |  |  |  |  |
| **Table S1 continued** | |  |  |  |  |  |  |  |  |  |  |  |
|  | **Belgium** |  |  | **Germany** |  |  | **Ireland** |  |  | **Italy** |  |  |
|  | **Healthcare utilisation** | **Unit cost (€)Ɨ** | **Ref** | **Healthcare utilisation** | **Unit cost (€)Ɨ** | **Ref** | **Healthcare utilisation** | **Unit cost (€)Ɨ** | **Ref** | **Healthcare utilisation** | **Unit cost (€)Ɨ** | **Ref** |
| **POSTNATAL CARE** | |  |  |  |  |  |  |  |  |  |  |  |
| **Contacts with healthcare providers** |  |  |  |  |  |  |  |  |  |  |  |  |
| Obstetrician WTC/WC | 1/3 | 24.71 |  | 1/3 | 28.18 |  | 0/2 | 173.87 |  | 1/3 | 76.52 |  |
| Paediatrician WTC/WC | 1/3 | 34.13 |  | 2/4 | 56.70/34.08 |  | 0/2 | 173.87 |  | 1/3 | 76.52 |  |
| General practitioner |  |  |  |  |  |  | 2*** | 33.05 |  |  |  |  |
| Public health nurse | 2 | 29.01 |  |  |  |  | 1 | 28.33 |  |  |  |  |
| Midwife |  |  |  | 10 | 33.88 |  |  |  |  | 1 | 28.42 |  |
| **Diagnostic tests** |  |  |  |  |  |  |  |  |  |  |  |  |
| Ultrasound scan (mother) |  |  |  |  |  |  |  |  |  | 1 | 103.85 |  |
| Neonatal hearing screening | 1 | 29.01 |  | 1 | 19.20 |  |  |  |  |  |  |  |
| **OTHER** |  |  |  |  |  |  |  |  |  |  |  |  |
| Educational classes (midwife) |  |  |  | 14 hours | 6.53 |  |  |  |  |  |  |  |
| Travel costs (regular) | 29 km | 0.25 |  | 38 km | 0.23 |  | 45 km | 0.30 |  | 33 km | 0.22 |  |
| Productivity loss mortality | Per day | 132.91 |  | Per day | 137.80 |  | Per day | 127.69 |  | Per day | 96.64 |  |

Ɨ Currency in 2016 euros (€)

ERCD, elective repeat caesarean delivery; WTC, without complications; WC, with complications; Ass, assumption

* included in pregnancy follow-up fee obstetrician

** including Venereal disease research laboratory test (VDRL), white blood cell count (WBC), WBC differential count, red blood cell count, haematocrit (Hct), haemoglobin (Hb), mean corpuscular volume (MCV), mean corpuscular haemoglobin (MCH), mean corpuscular haemoglobin concentration (MCHC), red cell distribution width (RDW), platelet count, mean platelet volume (MPV)

*** one two week check of the neonate and one combined visit mother and neonate

| **Table S2.** Model parameters and distributions probabilistic sensitivity analysis | | | | | | |
| --- | --- | --- | --- | --- | --- | --- |
| **Decision tree transition probabilities** | | | | | | |
|  | **Country** | **Mean** | **Alpha** | **Beta** | **Distribution** | **Ref** |
| VBAC-UR | All | 0.0054 | 398 | 73074 | Beta |  |
| VBAC-UVB | BE  DE  IE  IT | 0.6504  0.6549  0.5742  0.6232 |  |  | Dirichlet  Dirichlet  Dirichlet  Dirichlet |  |
| VBAC-IVB | BE  DE  IE  IT | 0.0796  0.0801  0.0858  0.0700 |  |  | Dirichlet  Dirichlet  Dirichlet  Dirichlet |  |
| VBAC-EC | BE  DE  IE  IT | 0.2700  0.2650  0.3400  0.3068 |  |  | Dirichlet  Dirichlet  Dirichlet  Dirichlet |  |
| ERCD |  | 0.9600 |  |  | Dirichlet |  |
| ERCD-UVB |  | 0.0300 |  |  | Dirichlet | Expert Opinion |
| ERCD-IVB |  | 0.0100 |  |  | Dirichlet | Expert Opinion |
| **Probabilities morbidity and mortality per arm** | | | | | | |
| **Maternal** | |  |  |  |  |  |
|  | | **Mean** | **Alpha** | **Beta** | **Distribution** | **Ref** |
| VBAC-UR | |  |  |  |  |  |
| Maternal morbidity | | 0.55315 |  |  |  |  |
| Hysterectomy | | 0.12768 | 349 | 2383 |  |  |
| Endometritis | | 0.12500 | 350 | 2449 |  |  |
| Blood transfusion | | 0.14286 | 343 | 2056 |  |  |
| Thrombotic events | | 0.00132 | 399 | 301955 |  |  |
| Operative injury | | 0.15630 | 337 | 1821 |  |  |
| Wound complication | | 0.00000 | 400 | 12799999 |  |  |
| Maternal mortality | | 0.00593 | 398 | 66627 |  |  |
| VBAC-UVB | |  |  |  |  |  |
| Maternal morbidity | | 0.03250 |  |  |  |  |
| Hysterectomy | | 0.00082 | 400 | 487348 | Beta |  |
| Endometritis | | 0.02709 | 389 | 13965 | Beta |  |
| Blood transfusion | | 0.00390 | 398 | 101771 | Beta |  |
| Thrombotic events | | 0.00034 | 400 | 1225039 | Beta |  |
| Operative injury | | 0.00000 | 400 | 11339999 | Beta |  |
| Wound complication | | 0.00035 | 400 | 1143473 | Beta |  |
| Maternal mortality | | 0.00002 | 400 | 21704599 | Beta |  |
|  | |  |  |  |  |  |
|  | |  |  |  |  |  |
|  | |  |  |  |  |  |
| **Table S2 continued** | |  |  |  |  |  |
|  | | **Mean** | **Alpha** | **Beta** | **Distribution** | **Ref** |
| VBAC-IVB | |  |  |  |  |  |
| Maternal morbidity | | 0.03250 |  |  |  |  |
| Hysterectomy | | 0.00082 | 400 | 487348 | Beta |  |
| Endometritis | | 0.02709 | 389 | 13965 | Beta |  |
| Blood transfusion | | 0.00390 | 398 | 101771 | Beta |  |
| Thrombotic events | | 0.00034 | 400 | 1225039 | Beta |  |
| Operative injury | | 0.00000 | 400 | 11339999 | Beta |  |
| Wound complication | | 0.00035 | 400 | 1143473 | Beta |  |
| Maternal mortality | | 0.00002 | 400 | 21704599 | Beta |  |
| VBAC-EC | |  |  |  |  |  |
| Maternal morbidity | | 0.10083 |  |  |  |  |
| Hysterectomy | | 0.00174 | 399 | 225927 | Beta |  |
| Endometritis | | 0.06643 | 373 | 5247 | Beta |  |
| Blood transfusion | | 0.00574 | 398 | 68878 | Beta |  |
| Thrombotic events | | 0.00161 | 399 | 247721 | Beta |  |
| Operative injury | | 0.01230 | 395 | 31724 | Beta |  |
| Wound complication | | 0.01300 | 395 | 29973 | Beta |  |
| Maternal mortality | | 0.00005 | 400 | 7999199 | Beta | * |
| ERCD-ERCD | |  |  |  |  |  |
| Maternal morbidity | | 0.03252 |  |  |  |  |
| Hysterectomy | | 0.00085 | 400 | 737877 | Beta |  |
| Endometritis | | 0.01756 | 393 | 21981 | Beta |  |
| Blood transfusion | | 0.00247 | 399 | 160609 | Beta |  |
| Thrombotic events | | 0.00087 | 400 | 460616 | Beta |  |
| Operative injury | | 0.00231 | 399 | 172360 | Beta |  |
| Wound complication | | 0.00846 | 397 | 46484 | Beta |  |
| Maternal mortality | | 0.00008 | 400 | 5206372 | Beta |  |
| ERCD-UVB | |  |  |  |  |  |
| Maternal morbidity | | 0.03250 |  |  |  |  |
| Hysterectomy | | 0.00082 | 400 | 487348 | Beta |  |
| Endometritis | | 0.02709 | 389 | 13965 | Beta |  |
| Blood transfusion | | 0.00390 | 398 | 101771 | Beta |  |
| Thrombotic events | | 0.00034 | 400 | 1225039 | Beta |  |
| Operative injury | | 0.00000 | 400 | 11339999 | Beta |  |
| Wound complication | | 0.00035 | 400 | 1143473 | Beta |  |
| Maternal mortality | | 0.00002 | 400 | 21704599 | Beta |  |
| ERCD-IVB | |  |  |  |  |  |
| Maternal morbidity | | 0.03250 |  |  |  |  |
| Hysterectomy | | 0.00082 | 400 | 487348 | Beta |  |
| Endometritis | | 0.02709 | 389 | 13965 | Beta |  |
| Blood transfusion | | 0.00390 | 398 | 101771 | Beta |  |
| Thrombotic events | | 0.00034 | 400 | 1225039 | Beta |  |
| Operative injury | | 0.00000 | 400 | 11339999 | Beta |  |
| **Table S2 continued** | |  |  |  |  |  |
|  | | **Mean** | **Alpha** | **Beta** | **Distribution** | **Ref** |
| Wound complication | | 0.00035 | 400 | 1143473 | Beta |  |
| Maternal mortality | | 0.00002 | 400 | 21704599 | Beta |  |
| **Neonatal** | |  |  |  |  |  |
|  | | **Mean** | **Alpha** | **Beta** | **Distribution** | **Ref** |
| VBAC-UR | |  |  |  |  |  |
| Neonatal morbidity | | 0.26604 |  |  |  |  |
| Hypoxic ischemic encephalopathy | | 0.03130 | 387 | 11991 | Beta |  |
| Cerebral palsy | | 0.00376 | 398 | 105697 | Beta |  |
| Sepsis | | 0.18750 | 325 | 1408 | Beta |  |
| Respiratory conditions | | 0.04348 | 383 | 8416 | Beta |  |
| Neonatal mortality | | 0.03551 | 386 | 10477 | Beta |  |
| VBAC-UVB | |  |  |  |  |  |
| Neonatal morbidity | | 0.05332 |  |  |  |  |
| Hypoxic ischemic encephalopathy | | 0.00035 | 400 | 1142056 | Beta |  |
| Cerebral palsy | | 0.00004 | 400 | 9523009 | Beta |  |
| Sepsis | | 0.03142 | 387 | 11890 | Beta |  |
| Respiratory conditions | | 0.02151 | 391 | 17817 | Beta |  |
| Neonatal mortality | | 0.00002 | 400 | 21467481 | Beta |  |
| VBAC-IVB | |  |  |  |  |  |
| Neonatal morbidity | | 0.05451 |  |  |  |  |
| Hypoxic ischemic encephalopathy | | 0.00035 | 400 | 1142056 | Beta |  |
| Cerebral palsy | | 0.00004 | 400 | 9523009 | Beta |  |
| Sepsis | | 0.03261 | 387 | 11479 | Beta |  |
| Respiratory conditions | | 0.02151 | 391 | 17817 | Beta |  |
| Neonatal mortality | | 0.00002 | 400 | 21467481 | Beta |  |
| VBAC-EC | |  |  |  |  |  |
| Neonatal morbidity | | 0.09744 |  |  |  |  |
| Hypoxic ischemic encephalopathy | | 0.00072 | 400 | 554755 | Beta |  |
| Cerebral palsy | | 0.00009 | 400 | 4628829 | Beta |  |
| Sepsis | | 0.06845 | 372 | 4895 | Beta |  |
| Respiratory conditions | | 0.02818 | 389 | 13697 | Beta |  |
| Neonatal mortality | | 0.00002 | 400 | 8192601 | Beta |  |
| ERCD-ERCD | |  |  |  |  |  |
| Neonatal morbidity | | 0.05132 |  |  |  |  |
| Hypoxic ischemic encephalopathy | | 0.00000 | 400 | 2080399999999999 | Beta |  |
| **Table S2 continued** | |  |  |  |  |  |
|  | | **Mean** | **Alpha** | **Beta** | **Distribution** | **Ref** |
| Cerebral palsy | | 0.00000 | 400 | 2080399999999999 | Beta |  |
| Sepsis | | 0.02682 | 389 | 14121 | Beta |  |
| Respiratory conditions | | 0.02450 | 390 | 15534 | Beta |  |
| Neonatal mortality | | 0.00000 | 400 | 8100298 | Beta |  |
| ERCD-UVB | |  |  |  |  |  |
| Neonatal morbidity | | 0.05332 |  |  |  |  |
| Hypoxic ischemic encephalopathy | | 0.00035 | 400 | 1142056 | Beta |  |
| Cerebral palsy | | 0.00004 | 400 | 9523009 | Beta |  |
| Sepsis | | 0.03142 | 387 | 11890 | Beta |  |
| Respiratory conditions | | 0.02151 | 391 | 17817 | Beta |  |
| Neonatal mortality | | 0.00002 | 400 | 21467481 | Beta |  |
| ERCD-IVB | |  |  |  |  |  |
| Neonatal morbidity | | 0.05451 |  |  |  |  |
| Hypoxic ischemic encephalopathy | | 0.00035 | 400 | 1142056 | Beta |  |
| Cerebral palsy | | 0.00004 | 400 | 9523009 | Beta |  |
| Sepsis | | 0.03261 | 387 | 11479 | Beta |  |
| Respiratory conditions | | 0.02151 | 391 | 17817 | Beta |  |
| Neonatal mortality | | 0.00002 | 400 | 21467481 | Beta |  |
| **Utilities** | | | | | | |
|  | **Country** | **Mean** | **Alpha** | **Beta** | **Distribution** | **Ref** |
|  | BE  DE  IE  IT | 0.98  0.99  0.99  0.99 | 7  3  4  3 | 0  0  0  0 | Beta  Beta  Beta  Beta |  |
| **Disutilities** | | | | | | |
|  | | **Mean** | **Alpha** | **Beta** | **Distribution** | **Ref** |
| Maternal | | | | | | |
| VBAC | | 0.41 | 236 | 339 | Beta |  |
| EC | | 0.58 | 167 | 121 | Beta |  |
| ERCD | | 0.58 | 167 | 121 | Beta |  |
| Hysterectomy | | 0.58 | 167 | 120 | Beta |  |
| Uterine rupture | | 0.58 | 167 | 120 | Beta |  |
| Endometritis | | 0.38 | 250 | 416 | Beta |  |
| Blood transfusion | | 0.41 | 234 | 333 | Beta |  |
| Thrombotic events | | 0.41 | 234 | 333 | Beta | Ass |
| Operative injury | | 0.53 | 189 | 170 | Beta |  |
| Wound complication | | 0.53 | 189 | 170 | Beta | Ass |
| **Table S2 continued** | |  |  |  |  |  |
| **Disutilities** | | | | | | |
|  | | **Mean** | **Alpha** | **Beta** | **Distribution** | **Ref** |
| Neonatal | | | | | | |
| Hypoxic ischemic encephalopathy | | 0.75 | 365 | 3869 | Beta |  |
| Cerebral palsy | | 0.53 | 189 | 169 | Beta |  |
| Sepsis | | 0.01 | 398 | 90110 | Beta |  |
| Respiratory conditions | | 0.01 | 400 | 405979 | Beta |  |
| **Costs** | |  |  |  |  |  |
|  | **Country** | **Cost (€)Ɨ** | **Alpha** | **Beta** | **Distribution** | **Ref** |
| Antenatal care: Intention VBAC | BE  DE  IE  IT | 535.08  791.00  1,656.49  1,017.01 | 400  400  400  400 | 1  2  4  3 | Gamma  Gamma  Gamma  Gamma | See table S1 |
| Antenatal care: Intention ERCD | BE  DE  IE  IT | 563.14  826.08  1,843.91  1,095.35 | 400  400  400  400 | 1  2  5  3 | Gamma  Gamma  Gamma  Gamma | See table S1 |
| VBAC-UR-WTC | BE  DE  IE  IT | 5,104.82  3,903.29  4,805.12  2,876.06 | 400  400  400  400 | 13  7  12  5 | Gamma  Gamma  Gamma  Gamma |  |
| VBAC-UR-WC | BE  DE  IE  IT | 8,261.31  3,903.29  6,242.01  2,876.06 | 400  400  400  400 | 21  8  16  7 | Gamma  Gamma  Gamma  Gamma |  |
| VBAC-UVB-WTC | BE  DE  IE  IT | 2,948.27  1,744.85  2,336.97  1,315.01 | 400  400  400  400 | 7  4  6  3 | Gamma  Gamma  Gamma  Gamma |  |
| VBAC-UVB-WC | BE  DE  IE  IT | 3,362.94  2,394.32  4,065.48  1,673.74 | 400  400  400  400 | 9  6  10  4 | Gamma  Gamma  Gamma  Gamma |  |
| VBAC-IVB-WTC | BE  DE  IE  IT | 3,362.94  2,048.58  3,063.49  2,955.67 | 400  400  400  400 | 8  5  8  7 | Gamma  Gamma  Gamma  Gamma |  |
| VBAC-IVB-WC | BE  DE  IE  IT | 4,789.56  3,063.49  4,065.48  2,955.67 | 400  400  400  400 | 12  8  10  7 | Gamma  Gamma  Gamma  Gamma |  |
|  |  |  |  |  |  |  |
|  |  |  |  |  |  |  |
|  |  |  |  |  |  |  |
| **Table S2 continued** | | | | | | |
|  | **Country** | **Cost (€)Ɨ** | **Alpha** | **Beta** | **Distribution** | **Ref** |
| EC-WTC | BE  DE  IE  IT | 5,104.82  3,660.95  4,805.12  2,162.73 | 400  400  400  400 | 13  9  12  5 | Gamma  Gamma  Gamma  Gamma |  |
| EC-WC | BE  DE  IE  IT | 8,261.31  3,903.29  6,242.01  2,276.06 | 400  400  400  400 | 21  10  16  7 | Gamma  Gamma  Gamma  Gamma |  |
| ERCD-ERCD-WTC | BE  DE  IE  IT | 4,082.77  2,659.28  4,805.12  2,162.73 | 400  400  400  400 | 10  7  12  5 | Gamma  Gamma  Gamma  Gamma |  |
| ERCD-ERCD-WC | BE  DE  IE  IT | 5,104.82  3,179.50  6,242.01  2,876.06 | 400  400  400  400 | 13  8  16  7 | Gamma  Gamma  Gamma  Gamma |  |
| VBAC-UVB-WTC | BE  DE  IE  IT | 2,948.27  1,744.85  2,336.97  1,315.01 | 400  400  400  400 | 7  4  6  3 | Gamma  Gamma  Gamma  Gamma |  |
| VBAC-UVB-WC | BE  DE  IE  IT | 3,362.94  2,394.32  4,065.48  1,673.74 | 400  400  400  400 | 9  6  10  4 | Gamma  Gamma  Gamma  Gamma |  |
| VBAC-IVB-WTC | BE  DE  IE  IT | 3,362.94  2,048.58  3,063.49  2,955.67 | 400  400  400  400 | 8  5  8  7 | Gamma  Gamma  Gamma  Gamma |  |
| VBAC-IVB-WC | BE  DE  IE  IT | 4,789.56  3,063.49  4,065.48  2,955.67 | 400  400  400  400 | 12  8  10  7 | Gamma  Gamma  Gamma  Gamma |  |
| UVD/IB-DEATH | BE  DE  IE  IT | 11,315.02  2,048.58  4,065.48  2,955.67 | 400  400  400  400 | 28  5  10  7 | Gamma  Gamma  Gamma  Gamma |  |
| UR/EC/ERCD-DEATH | BE  DE  IE  IT | 13,481.30  3,063.49  6,242.01  2,876.06 | 400  400  400  400 | 34  8  16  7 | Gamma  Gamma  Gamma  Gamma |  |
| Neonatal HIE/CP (six-week time horizon) | BE  DE  IE  IT | 12,492.19  7,586.86  10,275.20  4,051.51 | 400  400  400  400 | 31  19  26  10 | Gamma  Gamma  Gamma  Gamma |  |
|  |  |  |  |  |  |  |
|  |  |  |  |  |  |  |
| **Table S2 continued** | | | | | | |
|  | **Country** | **Cost (€)Ɨ** | **Alpha** | **Beta** | **Distribution** | **Ref** |
| Neonatal HIE (first two years) | BE  DE  IE  IT | 16,926.18  16,423.42  17,093.77  15,250.32 | 400  400  400  400 | 42  41  43  38 | Gamma  Gamma  Gamma  Gamma |  |
| Neonatal Cerebral Palsy (lifetime) | BE  DE  IE  IT | 846,309.14  821,171.24  854,688.44  762,516.15 | 400  400  400  400 | 2116  2053  2137  1906 | Gamma  Gamma  Gamma  Gamma |  |
| Neonatal sepsis | BE  DE  IE  IT | 7,350.96  19,413.05  15,408.26  4,051.51 | 400  400  400  400 | 18  49  39  10 | Gamma  Gamma  Gamma  Gamma |  |
| Neonatal respiratory distress syndrome | BE  DE  IE  IT | 11,392.07  19,413.05  15,408.26  13,478.28 | 400  400  400  400 | 28  49  39  34 | Gamma  Gamma  Gamma  Gamma |  |
| Neonatal transient tachypnoea | BE  DE  IE  IT | 3,006.68  788.41  1,485.33  578.93 | 400  400  400  400 | 8  2  4  1 | Gamma  Gamma  Gamma  Gamma |  |
| Neonatal mortality | BE  DE  IE  IT | 4,711.87  7,198.21  7,035.91  5,618.77 | 400  400  400  400 | 12  18  18  14 | Gamma  Gamma  Gamma  Gamma |  |
| Postnatal care WTC | BE  DE  IE  IT | 182.33  622.13  135.22  307.50 | 400  400  400  400 | 0  2  0  1 | Gamma  Gamma  Gamma  Gamma | See table S1 |
| Postnatal care WC | BE  DE  IE  IT | 329.18  781.66  884.92  643.17 | 400  400  400  400 | 1  2  2  2 | Gamma  Gamma  Gamma  Gamma | See table S1 |
| Productivity loss (per year) | BE  DE  IE  IT | 48,511.39  46,373.06  46,606.94  35,272.10 | 400  400  400  400 | 42  116  117  88 | Gamma  Gamma  Gamma  Gamma | See table S1 |

Ɨ Currency in 2016 euros (€)

VBAC, vaginal birth after caesarean; UR; uterine rupture, UVB, unassisted vaginal birth; IVB, instrumental vaginal birth; EC, emergency caesarean section; ERCD, elective repeat caesarean delivery; Ass, assumption; WTC, without complications; WC, with complications; HIE, hypoxic ischemic encephalopathy; CP, cerebral palsy

* Maternal death was 5 times higher for an EC compared with ERCD

| **Table S3.** Distribution mode of birth by country | | | | | | | | | | | | | | | | | | | | |
| --- | --- | --- | --- | --- | --- | --- | --- | --- | --- | --- | --- | --- | --- | --- | --- | --- | --- | --- | --- | --- |
|  | Intention VBAC (n)* | | | | | | | | | | | | | | | | | Intention ERCD (n)*^Ɨ^ | | |
|  | UR |  |  |  | UVB | | | | IVB | | | | EC | | | | ERCD | | UVB | IVB |
|  | BE | DE | IE | IT | BE | DE | IE | IT | BE | DE | IE | IT | BE | DE | IE | IT |  | |  |  |
| **Maternal outcome** |  |  |  |  |  |  |  |  |  |  |  |  |  |  |  |  |  | |  |  |
| **Well (no adverse outcomes)** | 120 | 120 | 120 | 120 | 31293 | 31507 | 28292 | 27220 | 3829 | 3855 | 3462 | 3331 | 12073 | 11849 | 15202 | 16320 | 46435 | | 1451 | 484 |
| **Total morbidity** | 148 | 148 | 148 | 148 | 1052 | 1058 | 951 | 915 | 129 | 130 | 116 | 112 | 1352 | 1329 | 1705 | 1830 | 1561 | | 49 | 16 |
| Hyster-ecomy | 34 | 34 | 34 | 34 | 27 | 27 | 24 | 23 | 3 | 3 | 3 | 3 | 23 | 23 | 30 | 32 | 41 | | 1 | 0 |
| Endo-metritis | 34 | 34 | 34 | 34 | 877 | 882 | 792 | 762 | 108 | 108 | 97 | 93 | 892 | 876 | 1123 | 1206 | 843 | | 41 | 14 |
| Blood transfusion | 38 | 38 | 38 | 38 | 126 | 127 | 114 | 110 | 16 | 16 | 14 | 14 | 77 | 76 | 97 | 104 | 119 | | 6 | 2 |
| Trombotic events | 0 | 0 | 0 | 0 | 11 | 11 | 11 | 10 | 1 | 2 | 1 | 1 | 21 | 21 | 27 | 29 | 41 | | 1 | 0 |
| Operative injury | 42 | 42 | 42 | 42 | 0 | 0 | 0 | 0 | 0 | 0 | 0 | 0 | 165 | 162 | 208 | 223 | 111 | | 0 | 0 |
| Wound compli-cation | 0 | 0 | 0 | 0 | 11 | 11 | 10 | 10 | 1 | 1 | 1 | 1 | 174 | 171 | 220 | 236 | 406 | | 0 | 0 |
| **Mortality** | 2 | 2 | 2 | 2 | 1 | 1 | 1 | 1 | 0 | 0 | 0 | 0 | 1 | 1 | 1 | 1 | 4 | | 0 | 0 |
| **Neonatal outcome** |  |  |  |  |  |  |  |  |  |  |  |  |  |  |  |  |  | |  |  |
| **Well (no adverse outcomes)** | 189 | 189 | 189 | 189 | 30620 | 30830 | 27683 | 26635 | 3742 | 3768 | 3383 | 3255 | 12118 | 11894 | 15260 | 16383 | 45536 | | 1420 | 473 |
| **Table S3 continued** | | | | | | | | | | | | | | | | | | | | |
|  | Intention VBAC (n) | | | | | | | | | | | | | | | | | Intention ERCD (n)* ^Ɨ^ | | |
|  | UR |  |  |  | UVB | | | | IVB | | | | EC | | | | ERCD | | UVB | IVB |
|  | BE | DE | IE | IT | BE | DE | IE | IT | BE | DE | IE | IT | BE | DE | IE | IT |  | |  |  |
| **Total morbidity** | 72 | 72 | 72 | 72 | 1725 | 1735 | 1560 | 1500 | 216 | 217 | 195 | 188 | 1308 | 1285 | 1648 | 1768 | 2464 | | 80 | 27 |
| HIE | 8 | 8 | 8 | 8 | 11 | 11 | 11 | 10 | 2 | 1 | 1 | 1 | 10 | 10 | 12 | 13 | 0 | | 1 | 0 |
| CP | 1 | 1 | 1 | 1 | 2 | 1 | 1 | 1 | 0 | 0 | 0 | 0 | 1 | 1 | 1 | 1 | 0 | | 0 | 0 |
| Sepsis | 51 | 51 | 51 | 51 | 1016 | 1023 | 919 | 884 | 129 | 130 | 117 | 113 | 919 | 902 | 1158 | 1242 | 1288 | | 47 | 16 |
| Respira-tory conditions | 12 | 12 | 12 | 12 | 696 | 700 | 629 | 605 | 85 | 86 | 77 | 74 | 378 | 372 | 477 | 512 | 1176 | | 32 | 11 |
| **Mortality** | 9 | 9 | 9 | 9 | 1 | 1 | 1 | 1 | 0 | 0 | 0 | 0 | 0 | 0 | 0 | 0 | 0 | | 0 | 0 |

VBAC, vaginal birth after caesarean; ERCD, elective repeat caesarean delivery; UR, uterine rupture; UVB, unassisted vaginal birth; IVB, instrumental vaginal birth; EC, emergency caesarean section; HIE. hypoxic ischemic encephalopathy; CP, cerebral palsy

* per 50,000 women VBAC/ERCD arm

^Ɨ^ comparator idem for all four countries

| **Figure S1.** Incremental Net Monetary Benefit (lifetime horizon) |
| --- |
| 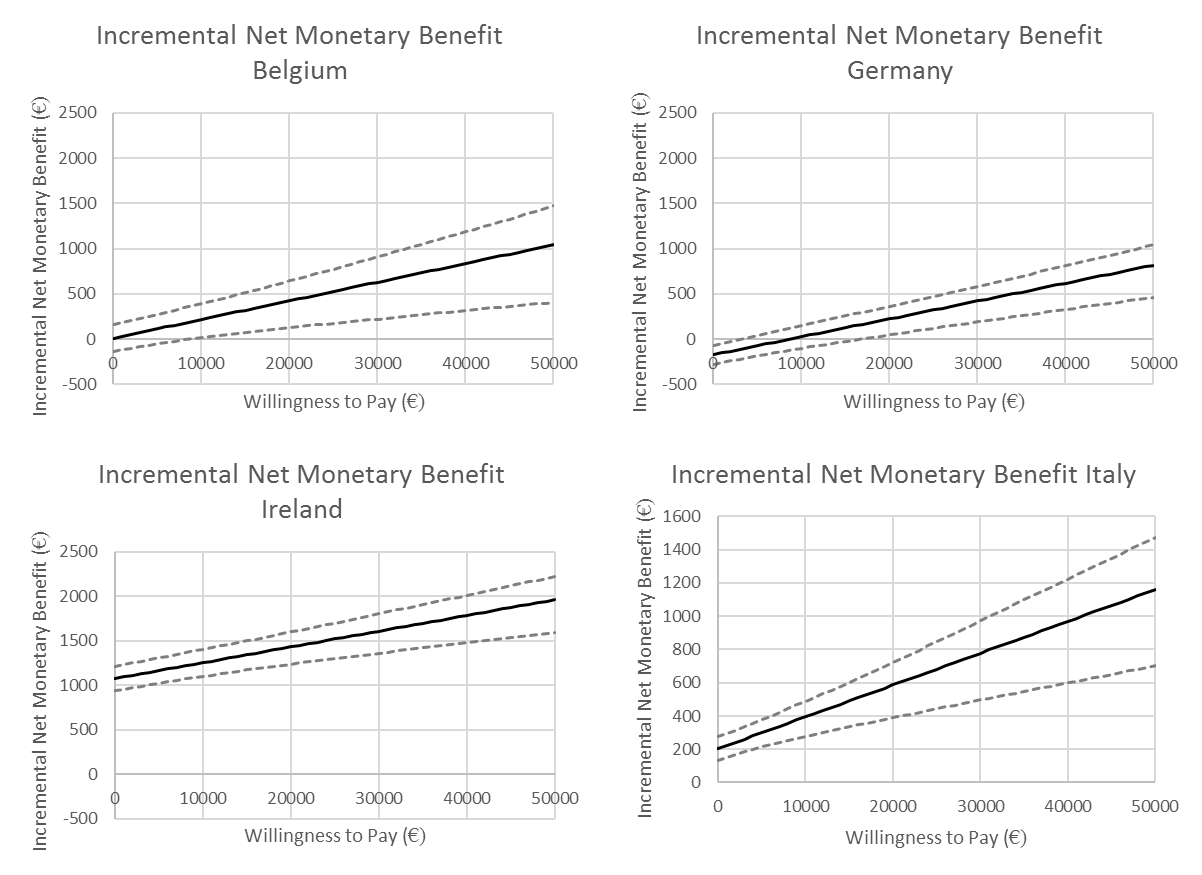 |
|  |

| **Figure S2.** Country specific tornado diagrams as a result of the one-way sensitivity analysis (six-week time horizon) |
| --- |
| 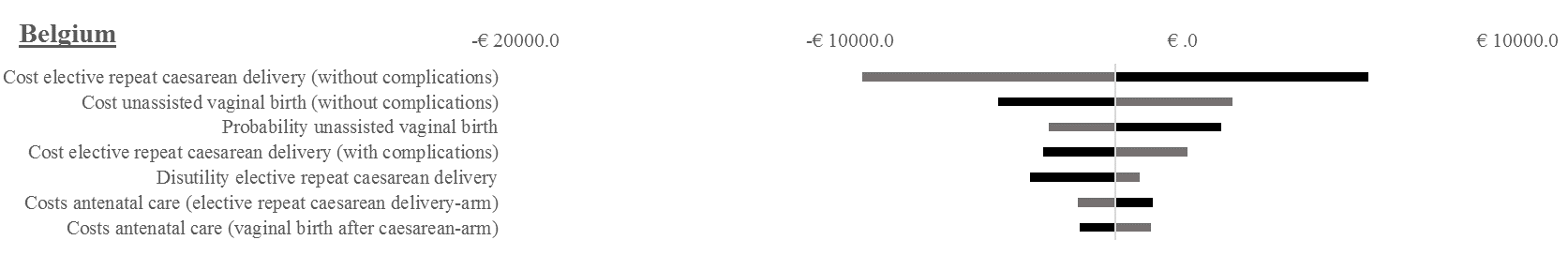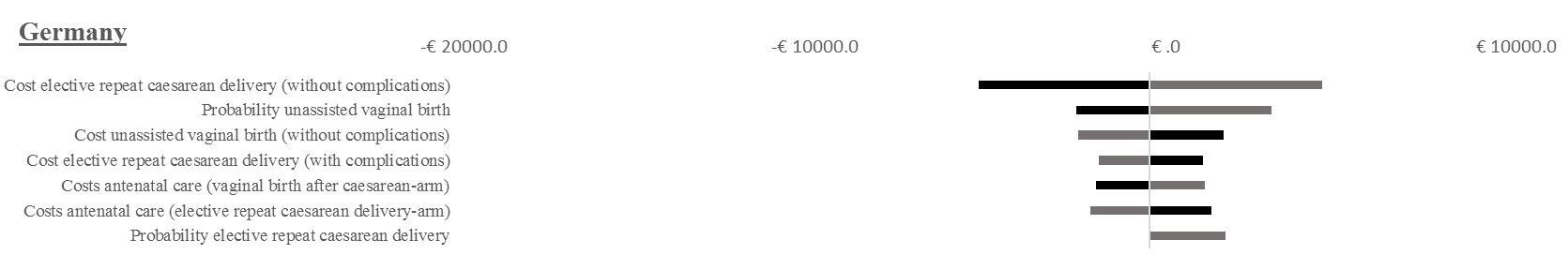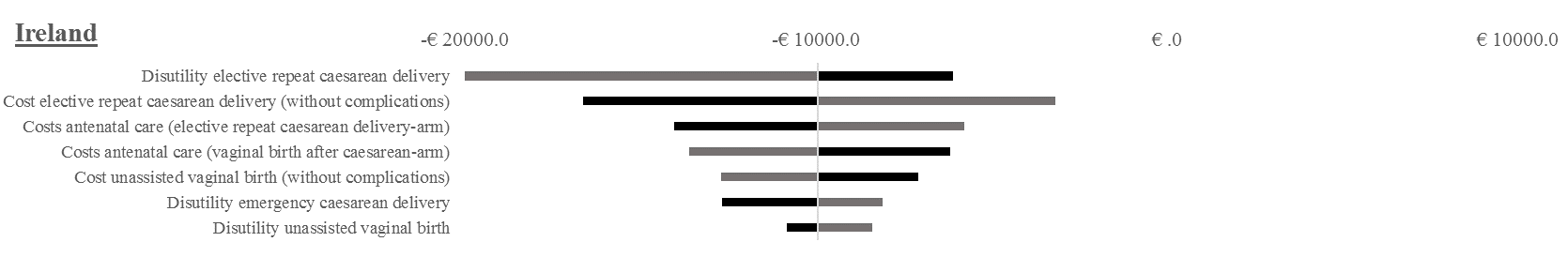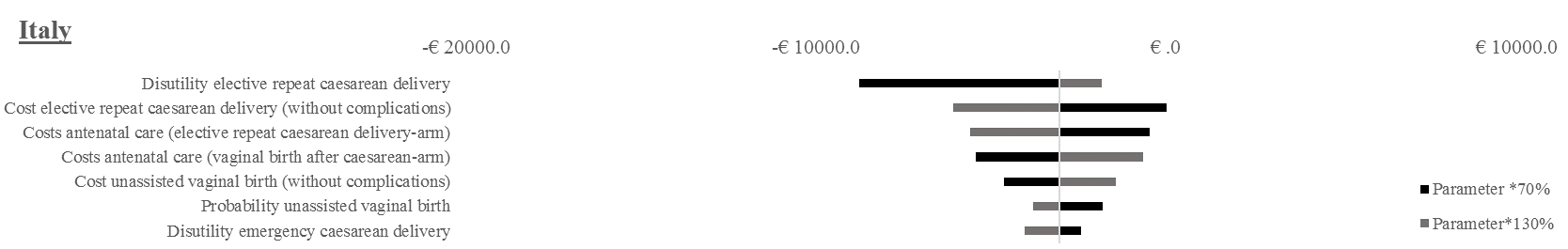 |
| ERCD, elective repeat caesarean delivery; VBAC, vaginal birth after caesarean; EC, elective repeat caesarean section; UVB, unassisted vaginal birth |

**References**
